# Egg yolk immunoglobulin interactions with *Porphyromonas gingivalis* to impact periodontal inflammation and halitosis

**DOI:** 10.1186/s13568-018-0706-0

**Published:** 2018-10-29

**Authors:** Wu Qiao, Fang Wang, Xiaochen Xu, Shujun Wang, Joe Mac Regenstein, Bin Bao, Ming Ma

**Affiliations:** 1 College of Food Science and Technology, Shanghai Ocean University, No. 999 Hucheng Ring Road, Pudong New Area, Shanghai, 201306 China; 2 Shanghai University of Medicine & Health Sciences, Shanghai, 201318 China; 3 Shanghai MAXAM Company Limited, Shanghai, 200333 China; 4 Co-Innovation Center of Jiangsu Marine Bio-industry Technology, Huaihai Institute of Technology, Lianyungang, 222005 China; 5 Department of Food Science, Cornell University, Ithaca, NY 14853-7201 USA; 6 Laboratory of Quality and Safety Risk Assessment for Aquatic Products on Storage and Preservation (Shanghai), Ministry of Agriculture, Shanghai, 201306 China

**Keywords:** Egg yolk immunoglobulin, *Porphyromonas gingivalis*, Periodontal inflammation, Halitosis, Volatile sulfur compounds, Volatile organic compounds

## Abstract

*Porphyromonas gingivalis* is a pathogenic Gram-negative anaerobic bacterium that colonizes the subgingival region of gums. These bacteria can invade periodontal tissues, form plaques, and produce volatile sulfur compounds (VSC) and volatile organic compounds (VOC). Egg yolk immunoglobulin (IgY) that was specifically produced in egg yolks after chickens were challenged with *P. gingivalis* could control and prevent oral diseases caused by *P. gingivalis*. The releases of *P. gingivalis* offensive metabolic odors in vitro and in vivo were determined using a Halimeter and GCMS. With IgY bacterial growth was inhibited, and the relative amounts of VOC and VSC were decreased. The scores for the oral health index and the levels of IL-6 and TNF-α are also decreased. All treatment groups showed significant anti-inflammatory effects, which strongly suggests that specifically IgY against *P. gingivalis* may be an effective treatment for the prevention and protection of periodontal inflammation and halitosis.

## Introduction

About 700 different microbial species cause a number of different infections and inflammation in the oral cavity (Kuramitsu et al. 2007; Tsuzukibashi et al. 2014). The most common microbial oral diseases are periodontal inflammation (Ke et al. 2016) and halitosis (Tanda et al. 2015). These diseases may be caused by various anaerobic bacteria such as *Streptococcus mutans*, *Porphyromonas gingivalis*, *Prevotella intermedia*, and *Fusobacterium nucleatum* (Zhen et al. 2008; Patra et al. 2014). *P. gingivalis* is one of the major pathogen that colonizes the subgingival region of gums. These bacterial can produce compounds that contribute to dental plaque, acquired immune response and release of unpleasant odor, such as protein adhesins, hemin binding proteins, proteinases, volatile sulfur compounds (VSC) and volatile organic compounds (VOC) (Holt et al. 1999; Lamont and Jenkinson 2000). The immune response results in tissue destruction and loss of alveolar bone and connective tissue, eventually leading to periodontal inflammation (Larsson 2017). VSC and VOC cause the unpleasant odors, which are referred to as halitosis (Lee et al. 2014).

Some approaches have been reported and applied to avoid oral diseases caused by *P. gingivalis*. They included inhibition of cell growth and initial cell adhesion, and degradation of odors emitted using antibacterial agents, and polyclonal and monoclonal antibodies. Honey as a therapeutic option in periodontitis treatment acted as an antibacterial against *P. gingivalis*, but did not inhibited the formation of biofilms in an in vitro study (Eick et al. 2014). *P. gingivalis* was found at a frequency of 35% in patients with chronic periodontitis and clinical isolates were highly sensitive to metronidazole and tetracycline (Gamboa et al. 2014). However, antibiotic resistance attributed to the use of metronidazole and tetracycline has become an increasing problem (Patil et al. 2013). Parenteral or intraoral administration of KAS2-A1-specific polyclonal antibodies protected against the development of *P. gingivalis*-induced bone resorption and inhibited proteolytic activity, binding to host cells/proteins and co-aggregation with other periodontal bacteria (O’Brien-Simpson et al. 2016). VSC were produced primarily by anaerobic bacteria that produced large amounts of proteinases, many trypsin-like (van den Velde et al. 2007). And oral malodors were reduced by inhibition of *P.* *gingivalis* growth and neutralization of VSC (Lee and Baek 2014). *Streptococcus thermophilus,* a probiotic bacterium, inhibited growth of *P. gingivalis*, and reduced *P. gingivalis*-producing VSC emissions. Nevertheless, *S. thermophilus* showed different antibacterial activity with different culture-conditions. A possible reason may be that *P. gingivalis* can inhibit the activity of antibacterial agents of *S. thermophilus* or has weak resistance for the antibacterial agent (Lee and Baek 2014).

Recently, chicken egg yolk immunoglobulin, referred to as immunoglobulin Y (IgY), has been shown to be a specific antibody that was produced in egg yolks after chickens were challenged with bacteria, viruses, or parasites. Thus, IgY could specifically control and prevent diseases caused by their corresponding antigen (Li et al. 2012). These may be a convenient and economical alternative to antibiotics to give passive immunization (Horie et al. 2004; Chalamaiah et al. 2017). IgY against *Pseudomonas aeruginosa* therapy facilitated rapid bacterial clearance and moderated inflammation in *P. aeruginosa* lung infections. It may serve as an adjunct to antibiotics in reducing early colonization (Thomsen et al. 2016). IgY against *Solobacterium moorei* significantly inhibited growth and biofilm formation of *S. moorei* and decreased the bacterial level in the oral cavity of mice after infection with *S. moorei* (Lee and Baek 2014). IgY against *P. gingivalis* inhibited dental plaque formation (Hamajima et al. 2007) and hemagglutinating activity of *P.* *gingivalis* (Tezuka et al. 2006) in vitro, which may be useful in developing passive immunization against periodontal diseases caused by *P. gingivalis* infection.

Because of rats’ periodontal anatomy similar to humans they are often used in the study of periodontal diseases (Helieh and David 2011). In addition, rodent models reflect the state of the periodontal more sensitively. Therefore, periodontal diseases commonly have been induced by placing a bacterial plaque retentive ligature in the gingival sulcus around the molar teeth in animal models (Breivik et al. 2000). The purpose of this study was to evaluate the effect of IgY against *P.* *gingivalis* on the growth of *P. gingivalis*, emission of VSC and VOC in vitro and in vivo, the scores for oral health indexes and the levels of IL-6 and TNF-α to determine if IgY might be an effective treatment for the prevention of periodontal inflammation and halitosis caused by *P. gingivalis*.

## Materials and methods

### Materials

This study used IgY against *P. gingivalis* (25,600 titer, 370 mmol/l, according to the manufacturer) from Maxam Ltd. (Shanghai, China), and *P. gingivalis* (ATCC33277) was purchased from American Type Culture Collection (Manassas, VA, USA). All other chemicals were analytical reagent grade or better from Chinese suppliers.

The simple preparing process of *P. gingivalis* as an antigen: *P. gingivalis* was maintained on Brain Heart Infusion Broth (BHI) supplemented with 10% sheep blood. Stable-phase *P. gingivalis* was dissolved in PBS (0.05 M, pH7.4) and stored at 4 °C. The solution mixed with Freund’s adjuvant in a ratio of 1:1 was used as the antigen.

### Growth inhibition

The artificial saliva was prepared using a method reported previously (Saunders et al. 2000; Bjorklund et al. 2011). Briefly, the artificial saliva was supplemented with mucin from porcine stomach (Sigma Chemical Co., St. Louis, MO, USA) 2500 mg/l. The saliva also contained KCl 1160 mg/l, NaHCO_3_ 375 mg/l, KH_2_PO_4_ 355 mg/l, NH_4_Cl 235 mg/l, KSCN 220 mg/l, CaCl_2_ 210 mg/l, urea 175 mg/l, MgCl_2_ 45 mg/l, thiamine 0.007 mg/l, riboflavin 0.05 mg/l, folic acid 0.0001 mg/l, nicotinic acid 0.03 mg/l, pyridoxine 0.6 mg/l, pantothenic acid 0.08 mg/l, biotin 0.0008 mg/l, B12 (cyanocobalamin) 0.003 mg/l, vitamin K (menaphthone) 0.015 mg/l, bovine serum albumin (Sinopharm Chemical Reagent Co., Shanghai, China) 25 mg/l, choline 15 mg/l, uric acid 10 mg/l, alanine 3.3 mg/l, arginine 1.9 mg/l, aspartic acid 1.6 mg/l, glutamic acid 3.9 mg/l, glycine 8.9 mg/l, histidine 1.0 mg/l, leucin 2.9 mg/l, iso-leucine 2.9 mg/l, lysine 2.7 mg/l, methionine 0.03 mg/l, phenylalanine 2.9 mg/l, proline 0.2 mg/l, serine 2.1 mg/l, threonine 2.9 mg/l, tyrosine 2.1 mg/l, valine 1.8 mg/l, creatinine 0.1 mg/l, and α-amylase 3 × 10^5^ U/l (Sigma). It was sterilized by passing through a 0.22 μm-filter (50 ml, Thermo Fisher Scientific, Shanghai, China). *P. gingivalis* were cultured in brain heart infusion broth (BHI) (Shandong Haibo Technology Information System Co., Ltd., Qingdao, Shandong, China). Colonies were counted using a hemocytometer (Shanghai Refined Biochemical Reagent Instrument Co., Ltd., Shanghai, China). Bacterial liquid (0.5 ml) was added to 4.5 ml artificial saliva at a final concentration of 10^8^ CFU/ml. Then 10 μl of IgY at concentrations of 370, 7.4 and 0.0037 mmol/l (diluted using 0.9% NaCl) was put into 5 ml tubes and were identified as the high-dose, mid-dose, and low-dose group, respectively. The mixtures without IgY were used as a control. These tubes were placed into an anaerobic package (Anaero Pack, Mitsubishi Gas Chemical Co., Tokyo, Japan), and incubated at 37 °C. OD_600_ of the suspensions was measured using a spectrophotometer (T6, Persee Ltd., Beijing, China) at 2 h intervals (Hou et al. 2014). The growth inhibition on *P. gingivalis* with IgY treatment was measured as OD_650_.

### Measurement of VSC in artificial saliva using a Halimeter

A portable sulphide monitor (Halimeter, Interscan Co., Chatsworth, CA, USA) was used to assess the VSC levels. Samples in sequence connect to the sampling tube of the Halimeter for 30 s according to the manufacturer’s instructions and three different readings were recorded per sample from the instrument without external calibration.

### Measurement of VOC in artificial saliva using GCMS

The artificial saliva was concentrated by putting 6 ml on a Poly-Sery Hydrophile Lipophilic Balance Solid Phase Extraction Cartridge (500 mg, Anpel, Shanghai, China) followed by passing dry nitrogen (99.9%, Linde, Shanghai, China) through the cartridge for 2 min. The sample was eluted with 1 ml methanol.

The gas chromatograph was a model: HP-6890N, interfaced with a mass selective detector HP-5973 (Agilent, Santa Clara, CA, USA). A fused silica capillary column HP-5MS (60 m × 0.32 mm × 1 μm, Agilent) was used with helium (99.999%, Linde) as the carrier gas (1 ml/min). The injection volume for each sample was 1 μl (Wang et al. 2018). The temperatures used were 250 °C for the detector, and 50–250 °C for the column with a heating rate of 8 °C/min. Peak identifications were done using the National Institute of Standards and Technology (NIST) electronic library (access provided by Agilent). The estimation of the approximate amounts of odor chemicals was based on the area under the peaks according to the software provided with the instruments and the determination of each peak’s proportional area of the total observed peak areas.

### Animal studies

Healthy male Wistar rats (n = 9, weight 160 ± 10 g) purchased from Slac Laboratory Animal Co., Ltd. (Shanghai, China) were housed 3/cage upon arrival in 3 standard ventilated cages for 1 week before experimental procedures began. All rats had ad libitum access to the rat feed and water throughout all experiments and were maintained on a 12 h light cycle with lights on at 8:00. The testing was performed between 8:00 and 16:00. The use of animals, ethical clearance, and the study protocol were duly approved by the U.K. Animals (Scientific Procedures) Act and User Committee of Shanghai ocean University (China). Maxillary second molars of the rats were ligated with 5-0 silk thread and inoculated with freshly prepared *P. gingivalis* (10^6^ CFU/ml). The rats were challenged with *P. gingivalis* eight times at 2-day intervals (Hou et al. 2014). The infected rats were randomly divided into three groups (n = 3). After the last infection, 6 rats were smeared using a syringe with different doses of 0.2 ml IgY against *P. gingivalis* solutions, and three rats served as a control group to which 0.2 ml non-specific IgY was applied each day in the oral cavity for up to 28 days. IgY at concentrations of 370 and 7.4 mmol/l (diluted using 0.9% NaCl) were identified as the high-dose and low-dose group, respectively.

### Measurement of VOC in the oral cavity using GCMS

The samples were obtained using swabs that were wiped for 30 s in the oral cavity. Then the swabs were placed at the bottom of a headspace vial with a screw top lid (20 ml, Supelco, Bellefonte, PA, USA) and extracted with a coated micro extraction fiber (65 μm, PDMS/DVB, Supelco) for 30 min at 65 °C. The desorption time of the extraction was 5 min according to the manufacturer’s instructions.

### Oral test

The plaque index (PI), gingival index (GI), and halitosis index (HI) were measured before and after treatment. The PI and GI were recorded using a periodontal probe (Pancer et al. 2016; Liu et al. 2012). Briefly, the periodontal pockets or gingival crevices of the selected tooth were probed and graded as follows: Score 0: Gingival margin and gingival papilla (GM&P) are healthy, and no bleeding is observed after a slight probe. Score 1: GM&P are mildly inflamed; and changes in color, absence of edema, and occurrence of punctate hemorrhages are observed after a slight probe. Score 2: GM&P are moderately inflamed; changes in color, severe edema, and bleeding are observed after a slight probe while the blood is still in the gingival crevice. Score 3: GM&P are severely inflamed; changes in color, severe edema, ulcer, and bleeding are observed after a slight probe; or spontaneous bleeding, and blood is observed flowing out of the gingival crevice. The HI was assessed by direct sniffing (score 0–4) done by only one person, and the scores reflected the degree of the odor (Hu et al. 2007).

### IL-6 and TNF-α assays in serum

The blood samples were taken from the heart after cervical dislocation. The serum was cleared from cellular components of the blood by centrifugation at 12,000 rpm for 10 min at 4 °C and stored at − 80 °C until use. IL-6 and TNF-α levels were measured using an enzyme-linked immunosorbent assay following the manufacturer’s instructions (Nanjing Jiancheng Bioengineering Institute, Nanjing, China). A blank hole without sample only adding chromogenic agent and terminated liquid were used for zero. Optical density (OD) at 450 nm was read using a Microplate Reader (Corona Electric, Hitachinaka, Japan).

### Statistical analysis

Statistical analysis was done using SPSS 11.0 for Windows (SPSS, Chicago, IL, USA). Data were shown as mean ± standard deviation. The student t-test was used to quantify data for the microbial experiments. While Mann–Whitney U test (a non-parametric statistical) analysis was used to quantify data for the animals experiments. The differences were considered significant at the level of *p* < 0.05.

## Results

### Effect of IgY on growth inhibition

**Fig. 1:**
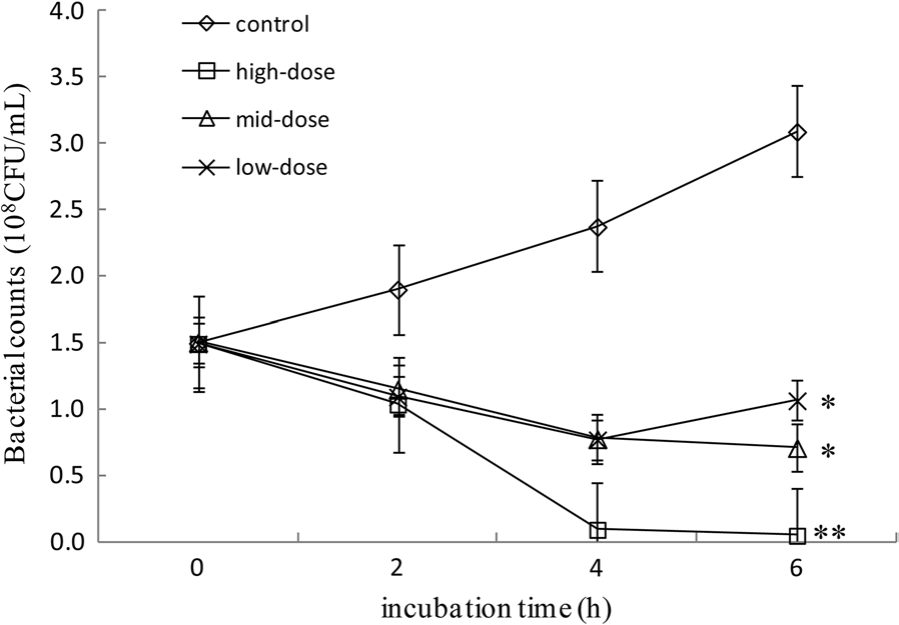
The inhibitory effect of IgY on the growth of *P. gingivalis*. *P. gingivalis* was cultured in artificial saliva medium containing 0 (control), 0.0037 (low-dose), 7.4 (mid-dose) or 370 (high-dose) mmol/l of IgY. Data were obtained using three independent experiments with three measurements in each experiment, and are shown as mean ± SD (n = 3). *Means different at *p *< 0.05, and **Means different at *p* < 0.01. *p* = 0.038 vs. the control and low-dose, *p* = 0.016 vs. the control and middle-dose, *p* = 0.008 vs. the control and high-dose

Upon addition of IgY in artificial saliva, bacterial cells formed complexes with IgY that precipitated to the bottom of the culture tubes, and the artificial saliva became clear (Zhen et al. 2008). Bacterial counts increased rapidly in the control (Fig. 1). However, the growth rate was significantly (*p* < 0.05) inhibited by the addition of IgY with each response significantly stronger than from the previous level.

### Effect of IgY on VSC inhibition

**Fig. 2:**
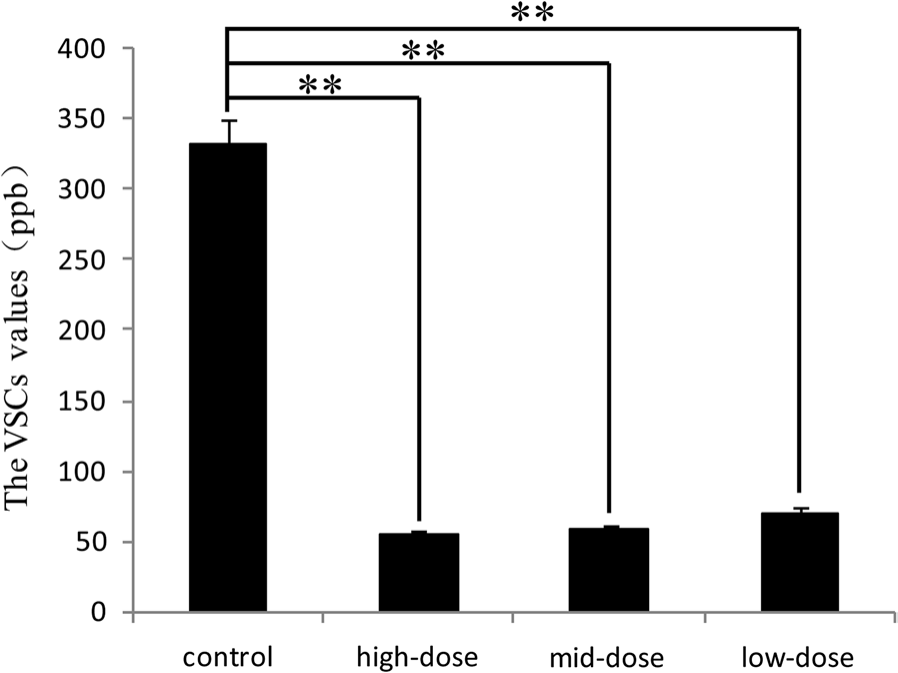
The VSC values of artificial saliva. The VSC values were recorded directly as the readings from the Halimeter, and the unit is ppb (ug/l). **Means different at *p* < 0.01. *p* = 0.007 vs. the control and low-dose, *p* = 0.006 vs. the control and middle-dose, *p* = 0.003 vs. the control and high-dose

The VSC values are shown in Fig. 2. All three doses reduce VSC significantly (*p* < 0.05) from the control but not significantly different from each other (*p* ≥ 0.05).

### Effect of IgY on VOC inhibition in artificial saliva

**Fig. 3:**
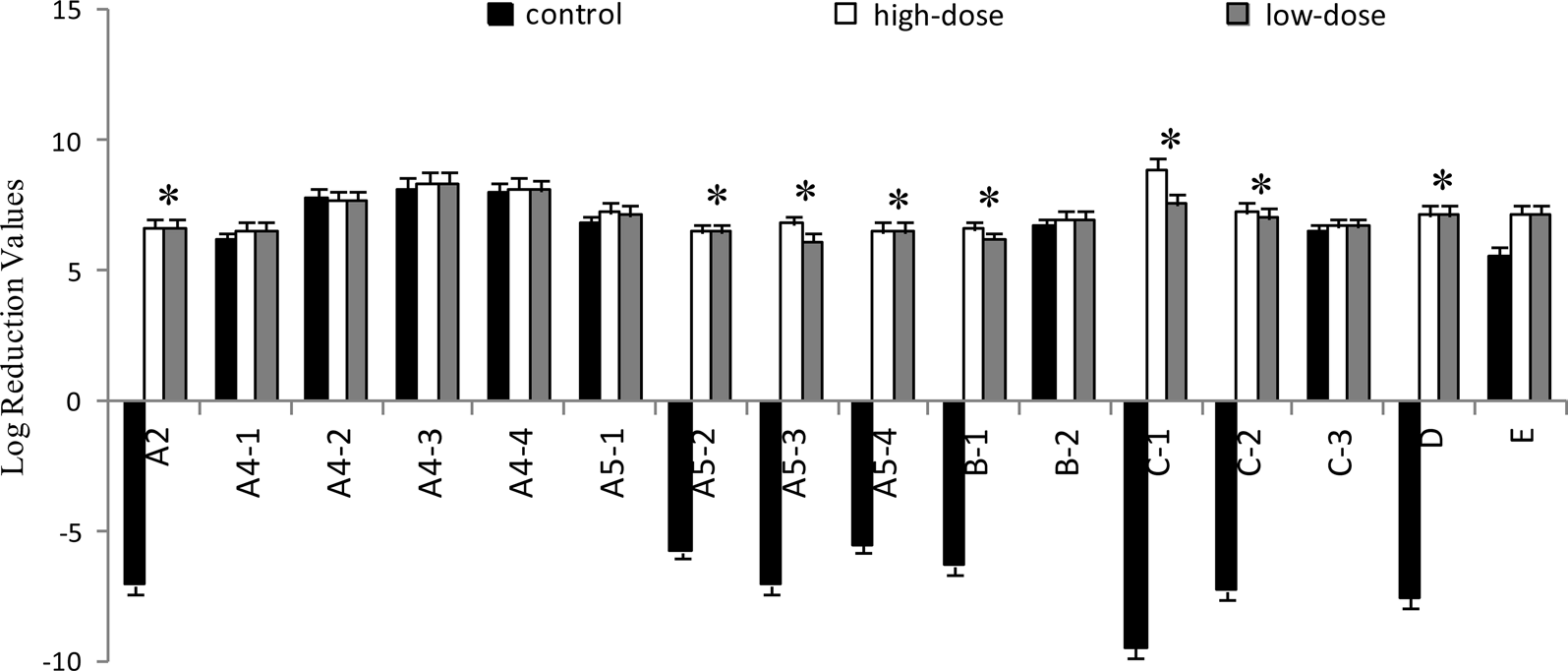
The reduction of VOC in artificial saliva. In artificial saliva 16 chemicals were detected (Table 1) using GCMS. Negative numbers represent an increase. The contents of VOC reduction are shown as mean with SD error bars (n = 3). *Means that the contents of the control group continued to increase, while the contents of the treatment group decreased significantly after administration. The *p* values are, in order, 0.027, 0.042, 0.033, 0.036, 0.034, 0.021, 0.030, 0.027 compared the control with the treatment groups

**Fig. 4:**
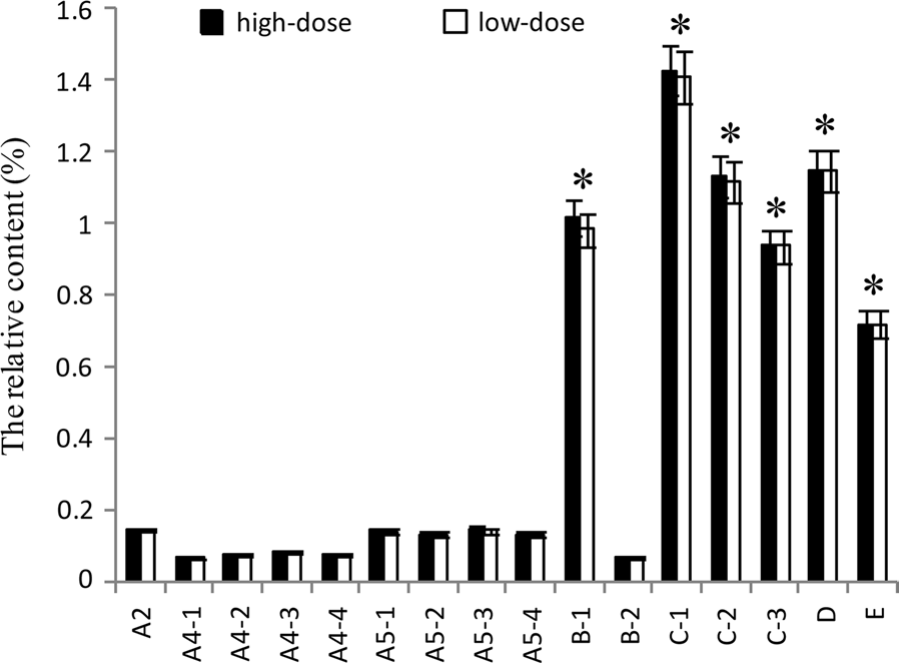
The proportional reduction of VOC with artificial saliva. According to distribute the proportion (Table 1), 16 chemicals were multiplied by corresponding proportion, and results are shown as mean ± SD (n = 3). *Means that the difference values between the treatment and control groups are more significant compare with another chemicals. The *p* values are, in order, 0.026, 0.017, 0.023, 0.031, 0.027, 0.035

VOC are products of *P. gingivalis* metabolism. The major classes produced are: alcohols (A1), aldehydes (A2), ketones (A3), carboxylic acids (A4), esters (A5), phenyl compounds (B), nitrogen compounds (C), sulfur-containing compounds (D), and alkanes (E) (Xia et al. 2008). In the artificial saliva, 16 chemicals were detected (Table 1). The relative contents of VOC were reduced before and after treatment with IgY (Fig. 3). The reduction of hydroxylamine was highest compared with control. The reductions of 3-bromo-5-chloro-1-tosyl-1H-pyrrolo[2,3-b] pyridine, benzeneacetaldehyde, 1,4-dioxoperhydropyrrolo[1,2-a]pyrazine, methyl elaidate, 2,4-Di-tert-butylphenol, methyl hexadecanoate, and methyl stearate were also significant (*p* < 0.05). The difference values between the control and treatment groups were multiplied by the corresponding proportion (Table 1, Fig. 4). The proportion of VOC were different depending on themselves odor (Outhouse et al. 2006). The relative content of 2,4-di-tert-butylphenol, hydroxylamine, 1,4-dioxoperhydropyrrolo [1,2-a] pyrazine, trans-13-docosenamide, 3-bromo-5-chloro-1-tosyl-1H-pyrrolo[2,3-b]pyridine, and undecane were also significantly reduced suggesting that IgY could reduce VOC by inhibiting bacterial growth.

**Table 1:** Chemical composition of VOC produced by *P. gingivalis* in artificial saliva detected using GCMS

No.	Retention time	CAS no	Name	Structure	Class	Proportion of odor (%)^a^
1	1.00–4.60	7803-49-8	Hydroxylamine		Nitrogen compounds (C-1)	15
2	10.8	122-78-1	Benzene acetaldehyde		Aldehydes (A2)	2
3	11.9	1120-21-4	Undecane		Alkanes (E)	10
4	19.3	96-76-4	2,4-Di-tert-butylphenol		Phenyl compounds (B-1)	15
5	22.9	19179-12-5	1,4-Dioxoperhydropyrrolo[1,2-a-pyrazine		Nitrogen compounds (C-2)	15
6	24.3	84-69-5	Diisobutyl phthalate		Phenyl compounds (B-2)	1
7	24.9	5129-60-2	Methyl 14-methylpentadecanoate		Esters (A5-1)	2
8	24.97	112-39-0	Methyl hexadecanoate		Esters (A5-2)	2
9	25	866-54-6	3-bromo-5-chloro-1-tosyl-1H-pyrrolo[2,3-b]pyridine		Sulfur-containing compounds (D)	15
10	25	2416-20-8	Hexadecenoic acid		Carboxylic acids (A4-1)	1
11	25.4	57-10-3	Hexadecanoic acid		Carboxylic acids (A4-2)	1
12	27	1937-62-8	Methyl elaidate		Esters (A5-3)	2
13	27.34	112-61-8	Methyl stearate		Esters (A5-4)	2
14	27.5	112-79-8	Elaidic acid		Carboxylic acids (A4-3)	1
15	27.7	57-11-4	Stearic acid		Carboxylic acids (A4-4)	1
16	29	10436-09-6	trans-13-docosenamide		Nitrogen compounds (C-3)	15

^a^According to the contribution to the VOC each chemical’s proportion represents its relative area under the peak without further calibration

### Effect of IgY on VOC inhibition in the oral cavity of rats

**Fig. 5:**
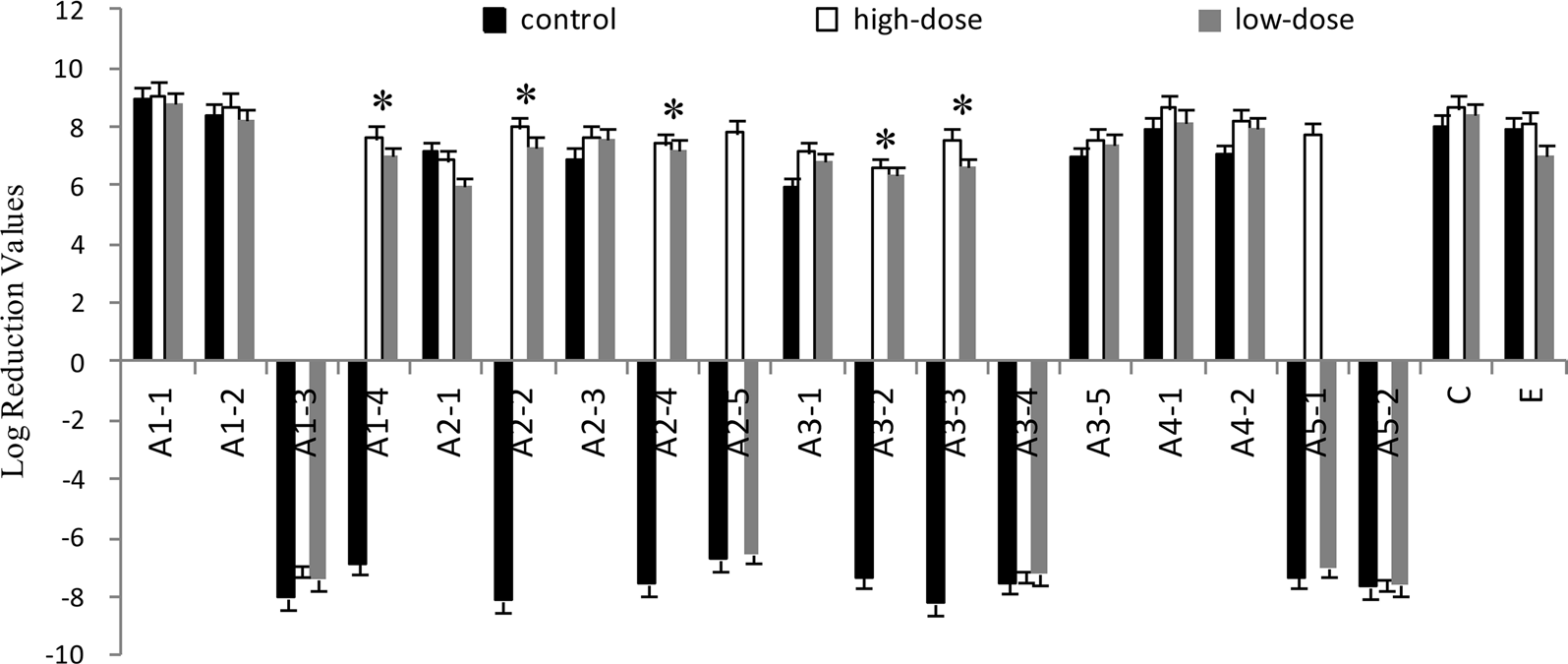
The reduction of VOC in the oral cavity of the rats. In the oral cavity of the rats, 20 chemicals were detected (Table 2) using GCMS. Negative numbers represent an increase. The contents of VOC reduction are shown as mean with SD error bars (n = 3). *Means that the contents of the control group continued to increase, while the contents of the treatment group decreased significantly after administration. The *p* values are, in order, 0.033, 0.028, 0.043, 0.026, 0.037 compared the control with the treatment groups

**Fig. 6:**
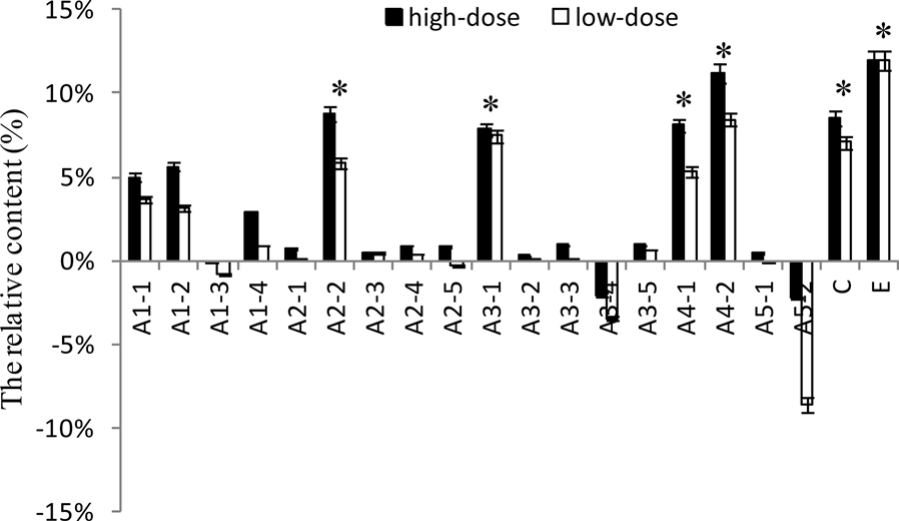
The reduction of VOC with a proportion in the oral cavity of the rats. Negative numbers represent an increase. According to distribute the proportion (Table 2), 16 chemicals were multiplied by corresponding proportion, and results are shown as mean ± SD (n = 3). *Means that the difference values between the treatment and control groups are more significant compare with another chemicals. The *p* values are, in order, 0.036, 0.022, 0.042, 0.035, 0.023, 0.018

Data from GCMS suggested that the bad breath of rats mainly was due to 9 classes of compounds with 20 specific chemicals involved (Table 2). After treatment with IgY for 4 wks, the relative contents of VOC were significantly reduced compared with control. The relatively higher reductions occurred with hexanal, 3,4,4-trimethylcyclopent-2-en-1-one, trans-2-octen-1-al, methyl isohexenyl ketone, and isooctyl alcohol (Fig. 5). The difference values between the control and treatment groups were multiplied by the corresponding proportion (Table 2, Fig. 6). The relative contents of hexanal, dimethylfuranone, acetic acid, hexanoic acid, ammonia, and 2-(vinyloxy) propane were significantly decreased (Fig. 6) suggesting that the odor compounds in the oral cavity are mainly composed of aldehydes, ketone, carboxylic acid, nitrogen compounds, and alkanes. The treatment with IgY can effectively inhibit the relative contents of these metabolites produced by *P. gingivalis*.

**Table 2:** Chemical composition of VOC produced by *P. gingivalis* in the oral cavity of the rats detected using GCMS

No.	Retention time	CAS no	Name	Structure	Classification	Proportion of odor (%)^a^
1	1.3	7664-41-7	Ammonia		Nitrogen compounds (C)	12
2	1.8	926-65-8	2-(Vinyloxy)propane		Alkanes (E)	12
3	2.5	64-19-7	Acetic acid		Carboxylic acids (A4-1)	12
4	4.3	100-50-5	Cyclohex-3-ene-1-carbaldehyde		Aldehydes (A2-1)	1
5	5.3	71-41-0	Pentanol		Alcohols (A1-1)	5
6	6.4	66-25-1	Hexanal		Aldehydes (A2-2)	12
7	8.1	35298-48-7	Dimethylfuranone		Ketones (A3-1)	12
8	9-10	111-27-3	*n*-Hexanol		Alcohols (A1-2	6
9	14.3	3391-86-4	1-Octen-3-ol		Alcohols (A1-3)	1
10	14.6	110-93-0	Methyl isohexenyl ketone		Ketones (A3-2)	1
11	15.2	124-13-0	Octyl aldehyde		Aldehydes (A2-3)	1
12	15.5	142-62-1	Hexanoic acid		Carboxylic acids (A4-2)	12
13	16.4	104-76-7	Isooctyl alcohol		Alcohols (A1-4)	6
14	17.3	695-06-7	gamma-hexalactone		Esters (A5-1)	1
15	17.4	2548-87-0	trans-2-Octen-1-al		Aldehydes (A2-4)	1
16	19.3	30434-65-2	3,4,4-Trimethylcyclopent-2-en-1-one		Ketones (A3-3)	1
17	23.1	112-31-2	Decyl aldehyde		Aldehydes (A2-5)	1
18	25.4	927-49-1	6-Undecanone		Ketones (A3-4)	1
19	26	540-07-8	Amyl caproate		Esters (A5-2)	1
20	28	6064-27-3	Dodecan-6-one		Ketones (A3-5)	1

^a^According to the contribution to the VOC each chemical’s proportion represents its relative area under the peak without further calibration

Microbial degradation is the main cause of oral malodor (Bollen and Beikler 2012). Combining the results of the Halimeter and GCMS assays in vitro and in vivo, the metabolites produced by *P. gingivalis* included VSC, aldehydes, ketones, carboxylic acids, esters, phenyl compounds, nitrogen compounds, and alkanes. After treatment with IgY, these metabolites were significantly reduced compared with control. Previous reports indicated that the main volatile molecules contributing to oral malodor included VSC, diamines, phenyl compounds, alcohols, alkanes, short-chain fatty acids, nitrogen compounds, and ketones (Bollen and Beikler 2012; Krespi et al. 2006). In addition, hexanal was detected in the experiment associated with halitosis. However, it is likely according to two reports that the hexanal may be a reaction intermediate. Because hexanal can generate hexanedione (Nomeir and Abou-Donia 1985) or hexanediol (Taher et al. 2009) with appropriate reaction conditions.

### Effect of IgY on oral health indexes

**Fig. 7:**
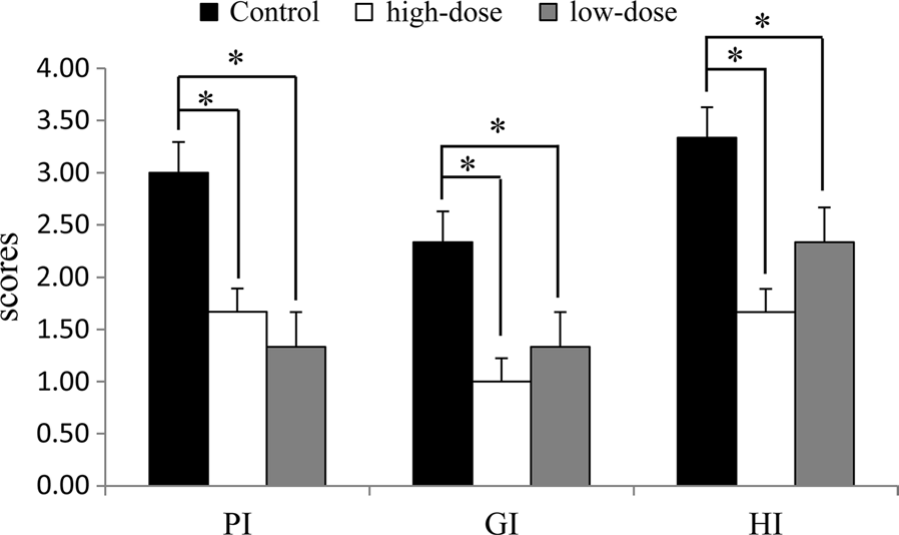
Effect of IgY on rat gingivitis and bad breath induced by *P. gingivalis*. The scores for oral health index are shown as mean ± SD (n = 3). *Means different at *p *< 0.05. For PI, *p* = 0.027, 0.025 compared the control with the high-dose and low-dose, respectively. For GI, *p* = 0.011, 0.032 compared the control with the high-dose and low-dose, respectively. For HI, *p* = 0.029, 0.036 compared the control with the high-dose and low-dose, respectively

The gingival of rats were red and swollen after 8 challenges with *P. gingivalis*, and the subgingival plaque and bad breath were obvious in most rats. The scores for GI, PI, and HI decreased significantly after treatment with IgY for 4 wks (Fig. 7). For PI, the three treatment groups’ scores were significantly lower than the control. For GI, the high-dose group’s scores were lower than the other treatment groups, and were higher than the positive control group. Thus, the scores of the high-dose group and positive control group were significantly decreased. For HI, the high-dose and mid-dose groups’ scores were significantly lower than control, while the low-dose group’s scores were higher than the positive control.

The PI and GI were used to diagnose periodontal disease and monitor traditional parameters (Bolerazska et al. 2016). The HI is an organoleptic parameter. The perception of odor is dependent of the olfactory response, the threshold concentration, the strength of the odor and the volatility of the molecules. When organoleptic scoring is done, a well-trained clinician determines if the odor samples smells bad or not, giving a score to the intensity (Hu et al. 2007). These oral health indicators in rats reflected the success of *P. gingivalis* infection (Pancer et al. 2016). After treatment with IgY for 4 weeks, these indicators showed statistically significant differences compared with controls, similar to previous research (Bostanci et al. 2013).

### Effect of IgY on levels of IL-6 and TNF-α in serum

**Fig. 8:**
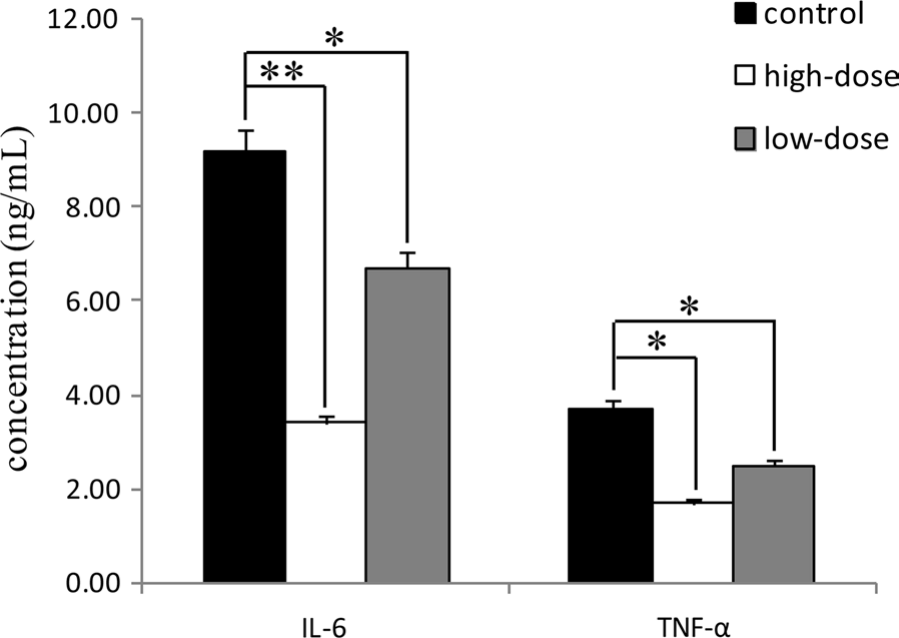
The levels of IL-6 and TNF-α in serum rats. The levels of IL-6 and TNF-α was measured using an ELISA Kit. Data are shown as mean ± SD (n = 3). *Means different at *p* < 0.05, **Means different at *p* < 0.01. For IL-6, *p* = 0.007, 0.038 compared the control with the high-dose and low-dose, respectively. For TNF-α, *p* = 0.032, 0.045 compared the control with the high-dose and low-dose, respectively

Abnormally high levels of inflammatory factors were seen in the control (Fig. 8). After treatment with IgY for 4 weeks, the levels of IL-6 and TNF-α in the low-dose group decreased significantly (*p* < 0.05). At the high-dose, the level of IL-6 was significantly (*p* < 0.01) lower as was the level of TNF-α (*p* < 0.05) compared to control. IL-6 and TNF-α in rat serum promote the production of inflammatory factors (Pan et al. 2017). These markers can be studied both in vitro and in vivo to learn more about the impact of the treatment. The experimental rats’ serum stimulated a pro-inflammatory response induced by infection of *P. gingivalis*, while the treatment with IgY decreased the levels of IL-6 and TNF-α and shifted the immune response towards an anti-inflammatory response (Bollen and Beikler 2012).

## Discussion

In the present study, IgY showed significant inhibitory effects on the bacterial growth of *P. gingivalis* (Fig. 1) and the production of bacterial metabolites under in vitro (Figs. 2, 3 and 4) and in vivo (Figs. 5 and 6) conditions, as well as moderate inhibitory effects on the development of periodontal inflammation and halitosis. IgY both reduced the growth speed of *P. gingivalis* and decreased the concentrations of their metabolites to a greater degree than non-specific IgY, whereas the clinical symptoms of periodontal inflammation and halitosis were also suppressed (Fig. 7). These results suggest that IgY has multiple suppressive effects on periodontal inflammation and halitosis induced by *P. gingivalis*.

The chronic periodontal inflammation appears to increase the patients’ risk for cardiovascular diseases (Genco and Van Dyke 2010), diabetes (Simpson et al. 2015), cancer (Babic et al. 2015), chronic respiratory diseases (Cardoso et al. 2018), adverse pregnancy outcomes (Parihar et al. 2015), and possibly rheumatoid arthritis (Kharlamova et al. 2016). Indeed, conventional periodontal treatment is often not enough by itself to treat destructive inflammation. Developing innovative adjunctive therapeutic strategies in chronic periodontitis is becoming an urgent need. Some projects have been successfully tested to inhibit periodontitis in animal models including counteracting immune evasion (McIntosh and Hajishengallis 2012) or anti-cytokine therapy (Cui et al. 2017). Another approach to treating periodontitis is to the use of agents that promote the resolution of inflammation by major periodontal pathogens.

Some experiments revealed specific IgY can promote the resolution of inflammation in the pathogens infection diseased. Specific anti-*Vibrio anguillarum* IgY administration in fish tissues could reduce bacterial number and inflammatory cytokine expression, and enhanced the phagocytic activity of macrophages against *V*. *anguillarum* in vitro (Li et al. 2014). In the present study, two inflammatory cytokine expressions also were inhibited in the serum of rats by IgY against *P. gingivalis* (Fig. 8). IgY against *Escherichia coli* inhibited the growth of the pathogen in a dose-dependent manner and enhanced the phagocytosis of *E. coli* (Zhen et al. 2008). In the cystic fibrosis (CF) setting, entering *Pseudomonas aeruginosa* may be rapidly killed due to IgY against *P. aeruginosa* heightened local host response, reimbursing the compromised mucosal immunity and provides CF patients with immediate resistance to infection that reduces early colonization with *P. aeruginosa* and hamper the destructive progression into chronic infection (Thomsen et al. 2016). Specific anti-*Staphylococcus aureus* IgY between clinical and experimental mastitis caused by *S. aureus* can improve the milk quality and decrease bacterial counts in milk, moreover, the cure rates of mastitis by *S. aureus* mastitis were higher than that by penicillin (Zhen et al. 2009).

IgY was a potential alternative for therapy of the pathogen infection disease, which can stop the major periodontal pathogen from breaking gingival tissue and promote the resolution of inflammation. In the in vitro study, IgY obtained from hens immunized with *Solobacterium moorei* inhibited the growth of *S*. *moorei* and biofilm formation, meanwhile, in the in vivo study, it had ability to decrease the level of bacteria in the oral cavity of mice (Li et al. 2012). The IgY against *P. gingivalis* 122k-hemagglutinin A, which was one of the virulence factors of *P. gingivalis* could be useful in assessing the treatment of periodontal diseases induced by *P. gingivalis* (Tagawa et al. 2004). Anti-130k haemagglutinin IgY significantly inhibited *P. gingivalis*-associated haemagglutinating activity (Hamajima et al. 2007). *P. gingivalis* were able to evade immune surveillance and avoid clearance, which facilitates local invasions of periodontal tissues; while the host also generated immune responses to the bacterial challenge, which contributed to a bactericidal effect but may also lead to periodontal destruction (Ke et al. 2016), which may develop into periodontitis and halitosis. Treatments of periodontitis and halitosis were based on mechanical debridement of infectious sites and pharmacological antibacterial therapy (Huang et al. 2013). The treatments were costly and time consuming for the patient. Within the limitations of the present study, in the treatment of oral diseases infected by these pathogens specific IgY against oral pathogen was shown to be an effective immunotherapeutic substance that *P. gingivalis* is rigidified by combining with IgY.

In most cases, halitosis comes from the mouth itself. About 700 species of microbes collectively populate all human mouths. Some studies had shown that the periodontal microflora could be involved in the formation of halitosis, as they had the necessary enzymes and would have access to sulfur-containing peptides and amino acids in vivo (Loesche and Kazor 2002). We also performed metabolomics evaluations to elucidate the molecular basis of the effects of IgY against *P. gingivalis*. In the presence of IgY, a large number of metabolites showed changed concentrations, especially those in the VSC and VOC biodegradation, which were significantly decreased in a dose-dependent manner. VSC are produced by *P. gingivalis* from sulphur amino acids (Bollen and Beikler 2012) in the artificial saliva. Some researchers have found that intraoral bacteria metabolise desquamated epithelial cells and blood cells, leading to the production of VSC from cysteine and methionine and thus increasing the VSC values parallel to an increase in the scores for PI and GI (Migliario and Rimondini 2011). Previous work showed that tongue cleaning could be used to reduce bacteria as a treatment for halitosis in adults (Outhouse et al. 2006). Essentially any oral site in which microbial accumulation and putrefaction could occur may be an origin for oral malodor (Krespi et al. 2006). The methods to reduce halitosis included mechanical and chemotherapeutical. Mechanical reduction of malodor and of the intraoral bacterial count may be achieved by disrupting the tongue and tooth surface biofilm, thus decreasing the production of VSC and other VOC (Aung et al. 2015). Another method, reduction of oral malodor may be aided by the use of active antimicrobial compounds which decrease the bacterial load and thus decrease the VSC and VOC production (Bollen and Beikler 2012). However, the reduction was nonspecific, in other words, all bacterial types were reduced. From an immunological point of view, IgY produced by *Helicobacter pylori* whole-cell lysate had been reported to specificity prevent *H. pylori* infection (Horie et al. 2004). It was demonstrated that IgY was highly specific and had a significant effectiveness against pathogen. The advantages of IgY in the application included non-invasive during production, inexpensive, convenience of animal handling and cost effective regarding the high IgY concentration within the egg yolk (Muller et al. 2015).

Although the animals and their surroundings are specific pathogen free, and the models of periodontal diseases are typical in this study. However, behavioral difference (e.g., exercise, diet, cleanliness) between individuals and variability in host responses to bacterial infection may still lead to increased standard deviation of results. Because of drinking and eating after administration, the duration of action of IgY and changes in the oral environments such as pH and salivation may also result in differences in the efficacy of IgY. Nonetheless, we always strictly control these influences during the experimental operation, and try to be uniformity.

In conclusion, with IgY bacterial growth was inhibited, and the relative contents of VOC and VSC were decreased in vitro and in vivo. The scores for the oral health indexes and the levels of IL-6 and TNF-α were also decreased in the animal experiments. All treatment groups showed significant anti-inflammatory effects suggesting that IgY may be an effective treatment for the prevention of periodontal inflammation and halitosis.

## Abbreviations

IgY: egg yolk immunoglobulin
VOC: volatile organic compounds
VSC: volatile sulfur compounds
GCMS: gas chromatography mass spectrometry
PI: plaque index
GI: gingival index
HI: halitosis index
IL-6: interleukin-6
TNF-α: tumor necrosis factor α
CF: cystic fibrosis

## References

[CR1] Aung EE, Ueno M, Zaitsu T, Furukawa S, Kawaguchi Y (2015). Effectiveness of three oral hygiene regimens on oral malodor reduction: a randomized clinical trial. Trials.

[CR2] Babic A, Poole EM, Terry KL, Cramer DW, Teles RP, Tworoger SS (2015). Periodontal bone loss and risk of epithelial ovarian cancer. Cancer Causes Control.

[CR3] Bjorklund M, Ouwehand AC, Forssten SD (2011). Improved artificial saliva for studying the cariogenic effect of carbohydrates. Curr Microbiol.

[CR4] Bolerazska B, Marekova M, Markovska N (2016). Trends in Laboratory Diagnostic Methods in Periodontology. Acta Medica (Hradec Kralove).

[CR5] Bollen CM, Beikler T (2012). Halitosis: the multidisciplinary approach. Int J Oral Sci.

[CR6] Bostanci N, Ozturk VO, Emingil G, Belibasakis GN (2013). Elevated oral and systemic levels of soluble triggering receptor expressed on myeloid cells-1 (sTREM-1) in periodontitis. J Dent Res.

[CR7] Breivik T, Opstad PK, Gjermo P, Thrane PS (2000). Effects of hypothalamic-pituitary-adrenal axis reactivity on periodontal tissue destruction in rats. Eur J Oral Sci.

[CR8] Cardoso EM, Reis C, Manzanares-Cespedes MC (2018). Chronic periodontitis, inflammatory cytokines, and interrelationship with other chronic diseases. Postgrad Med.

[CR9] Chalamaiah M, Esparza Y, Temelli F, Wu J (2017). Physicochemical and functional properties of livetins fraction from hen egg yolk. Food Biosci.

[CR10] Cui D, Lyu J, Li H, Lei L, Bian T, Li L, Yan F (2017). Human beta-defensin 3 inhibits periodontitis development by suppressing inflammatory responses in macrophages. Mol Immunol.

[CR11] Eick S, Schafer G, Kwiecinski J, Atrott J, Henle T, Pfister W (2014). Honey—a potential agent against *Porphyromonas gingivalis*: an in vitro study. BMC Oral Health.

[CR12] Gamboa F, Acosta A, Garcia DA, Velosa J, Araya N, Ledergerber R (2014). Occurrence of *Porphyromonas gingivalis* and its antibacterial susceptibility to metronidazole and tetracycline in patients with chronic periodontitis. Acta Odontol Latinoam.

[CR13] Genco RJ, Van Dyke TE (2010). Prevention: reducing the risk of CVD in patients with periodontitis. Nat Rev Cardiol.

[CR14] Hamajima S, Maruyama M, Hijiya T, Hatta H, Abiko Y (2007). Egg yolk-derived immunoglobulin (IgY) against *Porphyromonas gingivalis* 40-kDa outer membrane protein inhibits coaggregation activity. Arch Oral Biol.

[CR15] Helieh SO, David AP (2011). AnimalModels for periodontal disease. J Biomed Biotechnol.

[CR16] Holt SC, Kesavalu L, Walker S, Genco CA (1999). Virulence factors of Porphyromonas gingivalis. Periodontol.

[CR17] Horie K, Horie N, Abdou AM, Yang JO, Yun SS, Chun HN, Park CK, Kim M, Hatta H (2004). Suppressive effect of functional drinking yogurt containing specific egg yolk immunoglobulin on *Helicobacter pylori* in humans. J Dairy Sci.

[CR18] Hou YY, Zhen YH, Wang D, Zhu J, Sun DX, Liu XT, Wang HX, Liu Y, Long YY, Shu XH (2014). Protective effect of an egg yolk-derived immunoglobulin (IgY) against *Prevotella intermedia*-mediated gingivitis. J Appl Microbiol.

[CR19] Hu Y, Hu DY, Zheng LL, Lin JH (2007). Establishment of malodor model and its effects on identifying the halitosis-related bacteria. Hua Xi Kou Qiang Yi Xue Za Zhi.

[CR20] Huang S, Huang Q, Huang B, Lu F (2013). The effect of *Scutellaria baicalensis* Georgi on immune response in mouse model of experimental periodontitis. J Dental Sci.

[CR21] Ke X, Lei L, Li H, Li H, Yan F (2016). Manipulation of necroptosis by *Porphyromonas gingivalis* in periodontitis development. Mol Immunol.

[CR22] Kharlamova N, Jiang X, Sherina N, Potempa B, Israelsson L, Quirke AM, Eriksson K, Yucel-Lindberg T, Venables PJ, Potempa J, Alfredsson L, Lundberg K (2016). Antibodies to *Porphyromonas gingivalis* indicate interaction between oral infection, smoking, and risk genes in rheumatoid arthritis etiology. Arthritis Rheumatol.

[CR23] Krespi YP, Shrime MG, Kacker A (2006). The relationship between oral malodor and volatile sulfur compound-producing bacteria. Otolaryngol Head Neck Surg.

[CR24] Kuramitsu HK, He X, Lux R, Anderson MH, Shi W (2007). Interspecies interactions within oral microbial communities. Microbiol Mol Biol Rev.

[CR25] Lamont RJ, Jenkinson HF (2000). Subgingival colonization by *Porphyromonas gingivalis*. Oral Microbiol Immunol.

[CR26] Larsson L (2017). Current concepts of epigenetics and its role in periodontitis. Curr Oral Health Rep.

[CR27] Lee SH, Baek DH (2014). Effects of *Streptococcus thermophilus* on volatile sulfur compounds produced by *Porphyromonas gingivalis*. Arch Oral Biol.

[CR28] Lee YJ, Lee DH, Jeong KJ (2014). Enhanced production of human full-length immunoglobulin G1 in the periplasm of *Escherichia coli*. Appl Microbiol Biotechnol.

[CR29] Li X, Liu H, Xu Y, Xu F, Wang L, You J, Li S, Jin L (2012). Chicken egg yolk antibody (IgY) controls *Solobacterium moorei* under in vitro and in vivo conditions. Appl Biochem Biotechnol.

[CR30] Li CH, Lu XJ, Li DF, Chen J (2014). Passive protective effect of chicken egg yolk immunoglobulins against experimental *Vibrio anguillarum* infection in ayu (*Plecoglossus altivelis*). Fish Shellfish Immunol.

[CR31] Liu R, Li N, Liu N, Zhou X, Dong ZM, Wen XJ, Liu LC (2012). Effects of systemic ornidazole, systemic and local compound ornidazole and pefloxacin mesylate on experimental periodontitis in rats. Med Sci Monit.

[CR32] Loesche WJ, Kazor C (2002). Microbiology and treatment of halitosis. Periodontol.

[CR33] McIntosh ML, Hajishengallis G (2012). Inhibition of *Porphyromonas gingivalis*-induced periodontal bone loss by CXCR4 antagonist treatment. Mol Oral Microbiol.

[CR34] Migliario M, Rimondini L (2011). Oral and non oral diseases and conditions associated with bad breath. Minerva Stomatol.

[CR35] Muller S, Schubert A, Zajac J, Dyck T, Oelkrug C (2015). IgY antibodies in human nutrition for disease prevention. Nutr J.

[CR36] Nomeir AA, Abou-Donia MB (1985). Analysis of n-hexane, 2-hexanone, 2,5-hexanedione, and related chemicals by capillary gas chromatography and high-performance liquid chromatography. Anal Biochem.

[CR37] O’Brien-Simpson NM, Holden JA, Lenzo JC, Tan Y, Brammar GC, Walsh KA, Singleton W, Orth RKH, Slakeski N, Cross KJ, Darby IB, Becher D, Rowe T, Morelli AB, Hammet A, Nash A, Brown A, Ma B, Vingadassalom D, McCluskey J, Kleanthous H, Reynolds EC (2016). A therapeutic *Porphyromonas gingivalis* gingipain vaccine induces neutralising IgG1 antibodies that protect against experimental periodontitis. NPJ Vaccines.

[CR38] Outhouse TL, Al-Alawi R, Fedorowicz Z, Keenan JV (2006). Tongue scraping for treating halitosis. Cochrane Database Syst Rev.

[CR39] Pan LY, Chen YF, Li HC, Bi LM, Sun WJ, Sun GF, Zhang XF, Xu K, Feng DX (2017). Dachengqi decoction attenuates intestinal vascular endothelial injury in severe acute pancreatitis in vitro and in vivo. Cell Physiol Biochem.

[CR40] Pancer BA, Kott D, Sugai JV, Panagakos FS, Braun TM, Teles RP, Giannobile WV, Kinney JS (2016). Effects of triclosan on host response and microbial biomarkers during experimental gingivitis. J Clin Periodontol.

[CR41] Parihar AS, Katoch V, Rajguru SA, Rajpoot N, Singh P, Wakhle S (2015). Periodontal disease: a possible risk-factor for adverse pregnancy outcome. J Int Oral Health.

[CR42] Patil V, Mali R, Mali A (2013). Systemic anti-microbial agents used in periodontal therapy. J Indian Soc Periodontol.

[CR43] Patra JK, Kim ES, Oh K, Kim HJ, Kim Y, Baek KH (2014). Antibacterial effect of crude extract and metabolites of *Phytolacca americana* on pathogens responsible for periodontal inflammatory diseases and dental caries. BMC Complement Altern Med.

[CR44] Saunders KA, Greenman J, McKenzie C (2000). Ecological effects of triclosan and triclosan monophosphate on defined mixed cultures of oral species grown in continuous culture. J Antimicrob Chemother.

[CR45] Simpson TC, Weldon JC, Worthington HV, Needleman I, Wild SH, Moles DR, Stevenson B, Furness S, Iheozor-Ejiofor Z (2015). Treatment of periodontal disease for glycaemic control in people with diabetes mellitus. Cochrane Database Syst Rev.

[CR46] Tagawa H, Kiyama-Kishikawa M, Lee SY, Abiko Y (2004). Inhibition of hemagglutinating activity by monoclonal antibody against *Porphyromonas gingivalis* 40-kDa outer membrane protein. Hybrid Hybridomics.

[CR47] Taher D, Thibault ME, Di Mondo D, Jennings M, Schlaf M (2009). Acid-, water- and high-temperature-stable ruthenium complexes for the total catalytic deoxygenation of glycerol to propane. Chemistry.

[CR48] Tanda N, Hoshikawa Y, Ishida N, Sato T, Takahashi N, Hosokawa R, Koseki T (2015). Oral malodorous gases and oral microbiota: from halitosis to carcinogenesis. J Oral Biosci.

[CR49] Tezuka A, Hamajima S, Hatta H, Abiko Y (2006). Inhibition of *Porphyromonas gingivalis* hemagglutinating activity by IgY against a truncated HagA. J Oral Sci.

[CR50] Thomsen K, Christophersen L, Bjarnsholt T, Jensen PO, Moser C, Hoiby N (2016). Anti-*Pseudomonas aeruginosa* IgY antibodies augment bacterial clearance in a murine pneumonia model. J Cyst Fibros.

[CR51] Tsuzukibashi O, Uchibori S, Shinozaki-Kuwahara N, Kobayashi T, Takada K, Hirasawa M (2014). A selective medium for the isolation of *Corynebacterium species* in oral cavities. J Microbiol Methods.

[CR52] van den Velde S, Quirynen M, van Hee P, van Steenberghe D (2007). Halitosis associated volatiles in breath of healthy subjects. J Chromatogr B Analyt Technol Biomed Life Sci.

[CR53] Wang Y, Li X, Jiang Q, Sun H, Jiang J, Chen S, Guan Z, Fang W, Chen F (2018). GC–MS analysis of the volatile constituents in the leaves of 14 compositae plants. Molecules.

[CR54] Xia Y, Chi Y, Zhu Q (2008). Shi pin feng wei hua xue = Shipin fengwei huaxue.

[CR55] Zhen YH, Jin LJ, Guo J, Li XY, Lu YN, Chen J, Xu YP (2008). Characterization of specific egg yolk immunoglobulin (IgY) against mastitis-causing *Escherichia coli*. Vet Microbiol.

[CR56] Zhen YH, Jin LJ, Li XY, Guo J, Li Z, Zhang BJ, Fang R, Xu YP (2009). Efficacy of specific egg yolk immunoglobulin (IgY) to bovine mastitis caused by *Staphylococcus aureus*. Vet Microbiol.

